# Modulatory Effects of Antioxidant Supplementation on Serum Oxidative Stress Biomarkers MDA and T-AOC in Females with Unexplained Infertility

**DOI:** 10.3390/antiox15050611

**Published:** 2026-05-12

**Authors:** Gabija Didžiokaitė, Aida Kuznecovaitė, Margarita Žvirblė, Žilvinas Survila, Vita Pašukonienė, Violeta Kvedarienė

**Affiliations:** 1 Faculty of Medicine, Vilnius University, 03101 Vilnius, Lithuania; 2 Faculty of Medicine, Institute of Biomedical Science, Department of Pathology, Vilnius University, 03101 Vilnius, Lithuania; 3 National Cancer Institute, P. Baublio Str. 3B, 08406 Vilnius, Lithuania; margarita.zvirble@gmail.com (M.Ž.); vita.pasukoniene@nvi.lt (V.P.); 4 Institute of Biosciences, Life Sciences Center, Vilnius University, Saulėtekio av. 7, 10257 Vilnius, Lithuania; 5 Center of Innovative Allergology, 03101 Vilnius, Lithuania

**Keywords:** unexplained infertility, female reproductive health, oxidative stress, reactive oxygen species, MDA, T-AOC, antioxidant supplements, antioxidant therapy

## Abstract

Unexplained infertility (UI) continues to pose a diagnostic challenge, affecting a considerable proportion of reproductive-aged women. Increasing evidence suggests that oxidative stress (OS) may contribute to impaired female reproductive function. Malondialdehyde (MDA) is a lipid peroxidation marker, while total antioxidant capacity (T-AOC) reflects overall antioxidant defense. Evaluating these biomarkers may help to better understand the role of OS in UI and the potential benefit of antioxidant therapy. A prospective observational study included 30 women diagnosed with primary unexplained infertility. Serum levels of MDA and T-AOC were measured at baseline and after a period of antioxidant supplementation lasting 1–7 months (duration mode: 3 months). All participants received standardized antioxidant therapy consisting of vitamin E (50 mg/day), zinc (15 mg/day), coenzyme Q10 (15 mg/day), and selenium (70 µg/day). Participants with known causes of infertility were excluded. Nonparametric statistical tests were used to evaluate changes in oxidative stress markers before and after supplementation and to compare subgroups with and without comorbidities. Median baseline MDA concentration was 228.2 ng/mL and decreased significantly after antioxidant supplementation to 173.9 ng/mL (*p* < 0.001), with a reduction observed in 90% of participants. Median T-AOC increased slightly from 23.9 U/mL to 26.2 U/mL, but the change was not statistically significant (*p* = 0.735). No significant differences in oxidative stress markers were found between women with and without comorbidities, although higher baseline MDA levels were observed in participants with endometriosis (stage I–II). A significant decrease in MDA after supplementation was seen both in women with endometriosis (*p* = 0.005) and without it (*p* < 0.001). Women with unexplained infertility demonstrate biochemical evidence of oxidative stress, reflected by elevated MDA levels. Antioxidant supplementation was associated with a significant reduction in lipid peroxidation, suggesting a potential therapeutic role of antioxidants in UI. Combined assessment of MDA and T-AOC may provide useful insight into oxidative imbalance in infertility, although larger controlled studies are needed.

## 1. Introduction

Infertility represents a global concern and a growing public health priority [1]. It is estimated to affect 12.6% to 17.5% of couples globally [2]. Despite thorough evaluation of both partners, the cause of infertility remains unidentified in about 15% of cases, leading to a diagnosis of “infertility of unknown origin” or “unexplained infertility” (UI) [3]. Furthermore, a diagnosis of unexplained infertility (UI) leaves couples without the ability to pursue targeted interventions, as its management is primarily based on empirical approaches and there is currently no standardized algorithm to guide treatment in these cases.

Recent studies have explored a range of biomarkers in an effort to elucidate the underlying causes and pathophysiological mechanisms contributing to infertility in patients diagnosed with unexplained infertility [4,5]. Among these, a particularly important group includes markers of oxidative stress. Oxidative stress (OS) is a condition characterized by an imbalance between the formation of reactive oxygen species (ROS) and the body’s ability to neutralize them via antioxidant defenses [6]. This imbalance can disrupt normal cellular signaling and cause molecular damage [6], contributing to the development and progression of many diseases, including cardiovascular, neurodegenerative, metabolic, autoimmune, and oncological disorders [7,8,9,10]. Certain female reproductive disorders, including polycystic ovary syndrome (PCOS), endometriosis, and premature ovarian failure, have also been associated with elevated OS levels [11]. In these conditions, fertility may decrease due to specific mechanisms affecting the reproductive tract, such as ovulatory dysfunction, poor oocyte quality, or structural abnormalities. However, OS per se is also considered an independent pathophysiological factor that can directly contribute to the development of infertility [12,13].

OS can damage cellular DNA and promote lipid peroxidation [14,15]. In the female body, DNA fragmentation may impair oocyte quality or embryonic development, while lipid peroxidation can disrupt cell membrane integrity and, consequently, negatively affect important reproductive processes [16,17]. Therefore, maintaining appropriate levels of oxidative stress is an important factor for optimal female reproductive function [18].

Malondialdehyde (MDA) is one of the most commonly used biomarkers to assess the extent of lipid peroxidation and, consequently, the level of oxidative stress in the body [19]. This marker is formed as a result of polyunsaturated fatty acid degradation during lipid peroxidation [20]. Total antioxidant capacity (T-AOC) represents the overall efficiency of the body’s defense system against oxidative damage by encompassing the collective effects [21]. T-AOC and TAC are synonymous and refer to total antioxidant capacity. While T-AOC provides valuable insight into the general antioxidant status, it should be interpreted together with other biochemical parameters to obtain a more comprehensive understanding of oxidative stress [22]. Among the oxidative stress markers reported in the literature, MDA and T-AOC were selected for the present study because they were among the most frequently evaluated biomarkers in gynecologic and fertility research and, importantly, reflect two complementary dimensions of redox imbalance that are particularly relevant to unexplained infertility: lipid peroxidation-related oxidative damage and the overall antioxidant defense capacity [23,24,25]. By capturing distinct and complementary aspects of antioxidant capacity and oxidative damage, these biomarkers together provide valuable insights into the oxidative imbalance associated with infertility.

The aim of our study was to evaluate serum MDA and T-AOC levels in women with unexplained infertility (UI) and to assess the influence of targeted antioxidant therapy on MDA and T-AOC concentration and, consequently, on oxidative stress levels and antioxidant capacity in these women. We hypothesized that women with unexplained infertility would exhibit increased oxidative damage, reflected by higher serum MDA levels, together with reduced antioxidant defense, reflected by lower T-AOC levels, and that targeted antioxidant therapy would partially restore this imbalance by decreasing MDA and improving T-AOC values.

## 2. Materials and Methods

A prospective observational study was conducted including 30 patients with primary unexplained infertility, who were referred to the Centre of Innovative Allergology in Vilnius, Lithuania. The inclusion and exclusion criteria are presented in Table 1. The study focused on within-subject comparisons of oxidative stress biomarkers before and after antioxidant supplementation in women with unexplained infertility, rather than comparisons with a healthy reference population, and therefore no control group was included in the study design.

The study was reviewed and approved by the Vilnius Regional Biomedical Research Ethics Committee (approval number 2024/2-1558-1026). All participants provided written informed consent prior to inclusion in the study. The dataset included the participants’ demographic characteristics, serum concentrations of malondialdehyde (MDA) and total antioxidant capacity (T-AOC), as well as data regarding gynecological anamnesis and co-existing comorbidities. Gynecological anamnesis comprised information on previously diagnosed pathologies that could affect female fertility, as well as results of prior diagnostic tests for gynecological disorders. The dataset was additionally split into subgroups according to co-existing comorbidities.

Serum concentrations of malondialdehyde (MDA) and total antioxidant capacity (T-AOC) were measured at the participants’ initial visit. Subsequently, all participants received standardized recommendations for antioxidant supplementation. Antioxidant therapy recommendations included specified daily doses of vitamin E (50 mg), zinc (15 mg), coenzyme Q10 (15 mg), selenium (70 µg). The selected agents were chosen by the study’s researchers based on a literature review evaluating the possible effects of these specific antioxidants on female fertility outcomes. Participant adherence was monitored through periodic investigator-led assessments in conjunction with self-reported intake records. It was further verified that, throughout the study period, participants did not smoke, undergo surgical interventions, receive treatment for severe or acute medical conditions, start any new medications or experience other significant confounding factors that could materially influence oxidative stress parameters.

Following the supplementation period, serum MDA and T-AOC levels were reassessed. The duration of antioxidant intake ranged from 1 to 7 months (duration mode: 3 months), depending on each participant’s individual infertility treatment plan. If further interventions that could increase oxidative stress (such as ovarian stimulation) were scheduled, the follow-up blood sample was obtained before their initiation; consequently, these factors partially influenced the duration of the antioxidant supplementation period for individual participants.

Venous blood samples were collected via venipuncture into BD Vacutainer tubes without anticoagulant for serum preparation (BD Biosciences, San Jose, CA, USA). After clotting at room temperature, samples were centrifuged, and the separated serum was stored at −80 °C until analysis.

Serum MDA concentrations were quantified in duplicate using commercial ELISA kits (Thermo Fisher Scientific, Vienna, Austria, and Waltham, MA, USA, respectively) according to the manufacturers’ protocols. The minimum detectable concentration for MDA was 18.75 ng/mL. Optical density was measured at 450 nm using a BioTek ELx800 TM plate reader (BIO-Tek Instruments, Winooski, VT, USA), and concentrations of MDA were determined using a 4PL curve, derived from the standard curve generated with the standards provided in an assay kit.

Serum T-AOC levels were determined using a colorimetric assay kit (Thermo Fisher Scientific, Vienna, Austria) following the manufacturer’s instructions. The assay is based on the reduction of ferric (Fe^3+^) to ferrous (Fe^2+^) ions by antioxidants present in the sample, forming a stable Fe^2+^–phenanthroline complex that produces a color change measurable at 520 nm. Absorbance was recorded using a Varioskan LUX Multimode Microplate Reader (Thermo Fisher Scientific, USA). T-AOC values were calculated according to the kit protocol and expressed in U/mL, with a detection range of 0.62–190.43 U/mL. Serum MDA, and T-AOC concentrations were measured twice—before and after a period of antioxidant supplementation.

Statistical data analysis was performed to evaluate differences in oxidative stress markers across study groups and time points. The normality of data distribution was first assessed using the Shapiro–Wilk test. Since most variables did not follow a normal distribution, nonparametric statistical tests were applied. Changes in serum marker concentrations before and after antioxidant use were analyzed using a two-sided exact Wilcoxon signed-rank test, while comparisons between independent groups were conducted using the Mann–Whitney U test. The corresponding effect sizes (r), expressed as rank–biserial correlations, were calculated to estimate the magnitude of observed differences. Correlations were assessed using Spearman’s rank correlation coefficient. All analyses were carried out using Python (version 3.11.4; Python Software Foundation). A two-tailed *p*-value < 0.05 was considered statistically significant. Given the relatively small sample size, this study should be considered exploratory and hypothesis-generating.

## 3. Results

A total of 30 women with primary unexplained infertility (UI) referred to an outpatient clinic in Lithuania were included in the study. The average age of the subjects was 34.8 (SD ± 4.6) years with a minimum of 26 and a maximum of 43 years old. Fifteen (50%) of all the women reported having at least one comorbidity, while 20% of all the women had conditions with a possible autoimmune etiology, including autoimmune thyroiditis, rheumatoid arthritis, ulcerative colitis, celiac disease, rosacea, and type 2 diabetes or allergic diseases. The median baseline MDA (MDA1) of all the women diagnosed with unexplained infertility was 228.2 ng/mL and the median baseline T-AOC (T-AOC1) was 23.9, as shown in Table 2.

Serum oxidative stress marker levels were analyzed in 30 women with unexplained infertility (UI) before and after 1–7 months of antioxidant supplementation, with a mode supplementation duration of 3 months. We did not observe statistically significant differences in changes in serum T-AOC or MDA levels across groups stratified by supplementation duration. Kruskal–Wallis testing showed no significant between-group differences for T-AOC (*p* = 0.43) or MDA (*p* = 0.10) among women who used antioxidant supplementation for <3 months (n = 6), 3 months (n = 18), and >3 months (n = 6). Therefore, no conclusions can be drawn regarding duration–response relationships due to low sample size.

**Figure 1:**
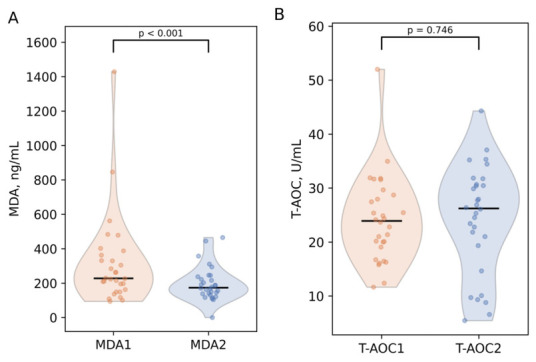
Serum MDA and T-AOC concentrations in infertile women before and after antioxidant use. (**A**) Changes in level of MDA, (**B**) changes in T-AOC.

Although no significant correlation was observed between the duration of antioxidant supplementation and change in serum MDA (ρ = −0.36, *p* = 0.053), T-AOC (ρ = −0.16, *p* = 0.409) levels, a significant decrease in MDA concentrations was observed after antioxidant intake, with median values declining from 228 (IQR: 171–355) ng/mL to 174 (IQR: 139–233) ng/mL (*p* < 0.001, Wilcoxon signed-rank test, *r* = 0.91) (Figure 1A). Serum MDA decreased for 27 (90%) of women with UI who took recommended dosages of antioxidants. Moreover, median serum T-AOC values showed a slight increase in the study group following antioxidant supplementation. Specifically, the median concentration rose from 23.9 U/mL (IQR: 19.3–28.5) at baseline to 26.2 U/mL (IQR: 19.8–30.6) post-supplementation (*p* = 0.735, r = 0.07) (Figure 1B). An increase in T-AOC levels was observed in 18 women (60%) after antioxidant use, indicating minimal response.

**Figure 2:**
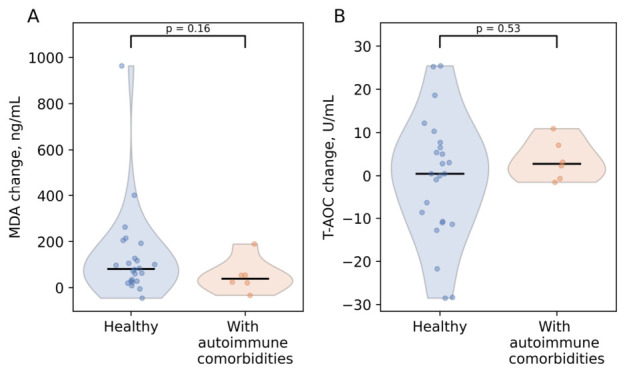
Changes in serum oxidative stress markers MDA and T-AOC in women with and without autoimmune conditions after antioxidant supplementation. (**A**) Changes in level of MDA, (**B**) changes in T-AOC.

When serum MDA and T-AOC levels before and after antioxidant supplementation were compared separately between women with any autoimmune conditions (N = 6) and women who did not report any autoimmune conditions (N = 24), an intriguing finding emerged: at baseline, median MDA level was lower in the first group (193.1 ng/mL, IQR: 143.4–252.0), compared with 246.6 ng/mL (IQR: 197.0–369.2) in the second group (*p* = 0.37; r = 0.25). Also, baseline T-AOC values before antioxidant supplementation were slightly higher in women with autoimmune conditions (24.0 U/mL, IQR: 23.0–25.1 vs. 22.8 U/mL, IQR: 16.7–29.0) (*p* = 0.67; r = 0.12). However, this difference in the medians of MDA and T-AOC values at baseline between healthy women and those with diseases did not reach a threshold of significance. Moreover, for both subgroups, a median decrease in MDA was observed after antioxidant supplementation. However, a moderately bigger decrease was observed for women without autoimmune conditions (80.9 ng/mL, IQR: 28.4–143.6) in comparison with women with autoimmune conditions (38.6 ng/mL, IQR: 21.2–53.7) (*p* = 0.16; r = 0.39). Minimal positive changes in total antioxidant capacity (T-AOC) after antioxidant supplementation were also observed for both groups. T-AOC levels were observed to increase slightly more for women with autoimmune conditions (median change of 2.8 U/mL, IQR: 0.1–6.1) in comparison with women without autoimmune conditions (median change of 0.5 U/mL, IQR: −10.8–6.8) (*p* = 0.53; r = −0.18) (Figure 2).

Nevertheless, when changes in serum MDA and T-AOC levels before and after antioxidant supplementation were analyzed separately within subgroups defined by specific comorbidities, no statistically significant differences were observed between women with individual comorbidities and otherwise healthy women (*p* > 0.05). As expected given the oxidative stress-driven nature of endometriosis, at baseline, median MDA levels were higher in women diagnosed with mild/moderate endometriosis (N = 13, 283.8 ng/mL IQR: 226.5–478.8) compared with those without this condition (N = 17, 210.4 ng/mL, IQR: 162.8–304.9).

In a separate analysis, participants were dichotomized based on the presence or absence of any comorbidity, regardless of type. Within this classification, no statistically significant differences in baseline oxidative stress markers were observed between women with at least one comorbidity and those without. Median MDA levels in women with chronic, autoimmune and gynecological comorbidities (N = 24) and without any comorbidities (N = 6) did not differ significantly (*p* = 0.56; r = 0.17). Similarly, baseline T-AOC values were comparable between women with and without comorbidities (*p* = 0.30; r = 0.29).

**Figure 3:**
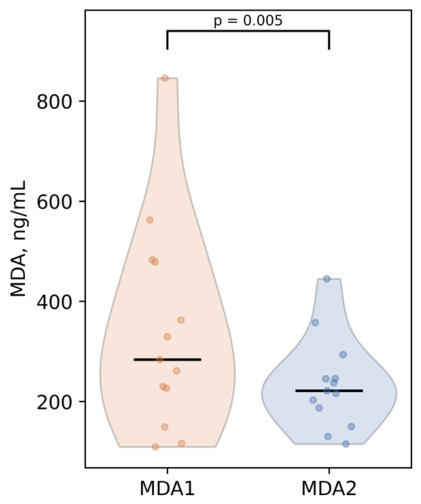
Changes in mean serum MDA levels after antioxidant supplementation in the endometriosis (stage I–II) group.

The study group was too small to determine subtle differences between the changes in OS levels after antioxidant intake for women who were diagnosed with specific chronic comorbidities separately, such as bronchial asthma, hypothyroidism or atopic dermatitis, as there were only a few cases of these conditions reported. However, for patients with mild/moderate endometriosis (N = 13), serum MDA levels statistically significantly decreased following the recommended antioxidant supplementation (*p* = 0.005, r = 0.85) with a similar significant reduction observed in endometriosis-free subjects (*p* = 0.0002, r = 0.94) (Figure 3). These findings highlight the beneficial effect of antioxidant therapy for this pathology, which is not only driven by OS, but also contributes to its further elevation as the disease progresses. However, in patients with endometriosis, final MDA levels remained higher (*p* = 0.02, r = 0.5) compared with those of endometriosis-free participants (median serum MDA 210.4 ng/mL (IQR: 162.8–304.9) vs. 283.8 ng/mL (IQR: 226.5–478.8)). This finding suggests that additional therapeutic strategies may be required to further reduce oxidative stress in individuals with this OS-related condition.

A decrease in serum MDA after antioxidant supplementation was also observed for almost all subgroups of women diagnosed with co-existing comorbidities, although the number of participants in the subgroups was insufficient to provide reliable statistical power.

Serum T-AOC did not differ significantly after antioxidant supplementation between any study subgroups (Table 3).

## 4. Discussion

### 4.1. Reactive Oxygen Species Impact on Female Reproductive Function

Reactive oxygen species (ROS) themselves can disrupt female reproductive function in several ways. OS can affect oocyte quality and folliculogenesis by impairing meiotic spindle formation and damaging mitochondria, thereby reducing fertilization potential [26,27,28]. The accumulation of ROS in cells damages mitochondrial DNA (mtDNA) and impairs mitochondrial function, resulting in decreased ATP synthesis. The resulting energy deficiency disturbs meiotic spindle formation, leading to improper chromosome segregation and oocyte developmental arrest [28]. Mitochondrial dysfunction and oxidative DNA damage in reproductive organs have been linked to implantation failure and early pregnancy loss [26,27,29].

OS can also adversely affect embryo development and implantation by inducing DNA and protein damage in early embryonic stages. Cooke et al. (2003) demonstrated that ROS interact with DNA bases to form free radical derivatives such as 8-hydroxyguanosine, leading to single- or double-strand DNA breaks [17]. If these lesions are not repaired, embryo development may arrest, implantation may fail, or miscarriage may occur [16,17]. Deluao et al. also reported that ROS can alter gene expression and epigenetic marks (e.g., DNA methylation), contributing to abnormal embryonic development and increased risk of congenital anomalies. Elevated ROS levels may disrupt early embryonic events—cell proliferation, cleavage, and blastocyst formation—while excessive DNA damage can activate apoptotic or autophagic processes, resulting in cell death and embryo loss [16].

Lipid peroxidation is the oxidative degradation of membrane lipids, especially polyunsaturated fatty acids, by ROS [30]. This process leads to the formation of toxic byproducts (e.g., malondialdehyde) that damage membrane structure [30]. Lipid peroxidation can disrupt uterine cell membrane integrity and reproductive processes by compromising endometrial receptivity and cell viability [31].

Oxidative stress (OS) is not only directly associated with female infertility but is also associated with specific gynecological diseases that can cause infertility, such as polycystic ovary syndrome (PCOS) and endometriosis [11]. In a literature review by Kaltsas et al. published in 2023, it is highlighted that OS likely contributes to the pathophysiology of PCOS, with factors such as insulin resistance and hyperandrogenism playing a role in increasing OS levels [11]. Also, increased levels of OS can disrupt ovarian follicle development, damage oocytes and granulosa cells, and impair ovulation, while hyperandrogenism and insulin resistance create a feedback loop that further elevates OS [32,33]. Several studies, including ours, have found elevated OS markers in women with endometriosis, such as higher levels of lipid peroxides, 8-OHdG (a marker of DNA damage), and altered antioxidant indicators in endometriosis cysts [34,35]. A theory regarding retrograde menstruation introducing blood, hemoglobin, and iron into the peritoneal cavity, promoting ROS production, is also suggested. This oxidative-favoring environment supports the survival, adhesion, and proliferation of ectopic endometrial tissue, partly by activating inflammatory and angiogenic pathways [36]. When the progression of endometriosis is promoted by the increase in OS levels, it can further increase ROS production, creating a cycle that not only worsens the extent of endometriosis itself, but also indirectly affects the processes of the female reproductive system by constantly increasing OS levels in the body [13].

### 4.2. Determinants of Oxidative Stress in Female Reproductive Health

Oxidative stress in women can arise due to both external and internal factors. External factors include poor diet, lifestyle habits such as smoking and alcohol consumption, exposure to environmental toxins, and natural aging processes [24,28,37,38]. Unbalanced nutrition and metabolic disorders (e.g., obesity or insulin resistance) also increase OS levels by inducing inflammation and mitochondrial dysfunction in reproductive tissues, disrupting the hormonal and oxidative balance necessary for fertility [37].

Agrawal et al. noted that both underweight and overweight conditions, as well as lifestyle habits such as smoking, alcohol, or drug use, enhance free radical production [24]. Zargari et al. (2022) found that environmental toxins—such as heavy metals like arsenic—can elevate OS in the reproductive system by stimulating excessive production of reactive oxygen and nitrogen species [38].

Aging in women is likewise associated with mitochondrial dysfunction and increased ROS accumulation in oocytes, resulting in poorer mitochondrial quality and a higher risk of chromosomal abnormalities [28]. The main factors driving this process include long-term accumulation of mitochondrial DNA (mtDNA) damage, impaired repair systems, and weakened antioxidant protection [39]. Over time, mtDNA mutations accumulate in oocyte mitochondria due to constant exposure to ROS—normal byproducts of mitochondrial energy production [39,40]. Oocytes have limited mtDNA repair capacity, particularly for double-strand breaks, allowing damage to persist [39,40]. Damaged mtDNA disrupts the mitochondrial respiratory chain, further increasing ROS generation and decreasing ATP synthesis [41].

Improving lifestyle quality is one of the key strategies to reduce OS levels. Kaltsas et al. emphasized that maintaining a healthy body weight, engaging in regular physical activity, and avoiding smoking, excessive alcohol consumption, and recreational drug use are associated with better oxidative balance and more favorable reproductive outcomes [11].

Tošić-Pajić et al. have also identified a link between OS and infertility caused by persistent Chlamydia infection, suggesting that timely treatment of sexually transmitted infections can help restore oxidative–antioxidative equilibrium [42]. Caturano et al. (2023) stressed that controlling blood sugar and diet directly influences OS levels and should also be considered as a part of infertility management [43]. Reddy et al. also noted that reducing the intake of advanced glycation end-products (AGEs), formed during high-temperature food processing, may decrease OS and related disease risk [7].

According to Al-Gubory et al., environmental pollution-induced OS can impair reproductive function and increase infertility risk [44]. External sources include heavy metals (e.g., mercury, cadmium, arsenic), pesticides, air pollutants (particulate matter, ozone), and industrial chemicals [45,46,47]. These compounds promote excessive ROS generation that exceeds the capacity of the body’s antioxidant systems, leading to DNA, protein, and lipid damage [45,47].

### 4.3. Malondialdehyde as an Oxidative Stress Biomarker

MDA is an oxidative damage marker corresponding to lipid peroxidation (LPO) levels. LPO damages cellular membranes, altering their permeability, fluidity, and integrity, and impairs the function of membrane-bound proteins and signaling pathways [48,49]. LPO generates toxic byproducts (e.g., malondialdehyde, 4-hydroxynonenal) that can further damage DNA, proteins, and other lipids, contributing to cell death (including ferroptosis) and inflammation [50,51]. Excessive LPO in the uterine epithelium disrupts redox balance, impairs uterine receptivity, and leads to implantation failure and pregnancy loss [31]. LPO also damages oocytes, causing meiotic errors and chromosomal misalignment [52]. Evidence supporting these mechanisms is derived from both experimental and limited clinical studies, and caution is warranted when extrapolating findings from non-human models to human reproductive outcomes. In 2020, Yang et al. showed in an in vivo *C. elegans* model that elevated lipid peroxidation is associated with germ cell loss, abnormal oogenesis, and impaired embryonic development [53]. Although these findings provide important mechanistic insights, they are derived from a non-human model and may not be directly translatable to human reproductive physiology.

Direct measurement of ROS is challenging due to their short lifespan and reactivity, so lipid peroxidation products serve as practical, quantifiable surrogates for oxidative stress [54]. MDA is one of the most frequently measured and accepted biomarkers for evaluation of oxidative stress, especially for assessing lipid peroxidation in both clinical and experimental settings [55].

### 4.4. MDA as a Marker of Oxidative Stress in Unexplained Infertility

In 2018, in a clinical study involving 60 women with unexplained infertility, Hussien et al. established that MDA levels were significantly higher in infertile patients compared to healthy controls, reflecting increased oxidative stress levels in women with unexplained infertility [56]. Moreover, in 2015, Soleimani Rad et al.’s comparative cross-sectional observational study also found a statistically significant increase in OS levels for women with UI: mean serum MDA of infertile women was 2.7 ± 0.47 nmol/mL while mean serum MDA for fertile controls was 1.91 ± 0.39 nmol/mL (*p* < 0.001) [57]. Similar findings were observed in our study, in which women with UI exhibited a mean serum MDA level of 4.37 ± 3.67 nmol/mL. Veena et al. (2008) reported mean serum MDA of 3.36 ± 0.16 nmol/mL in women with unexplained infertility versus 2.82 ± 0.15 nmol/mL in controls (*p* < 0.001) [25], although their Results section uses nmol/L and their Methods section uses nmol/mL, which aligns with our and other studies’ measurements [25]. In an analytical observational study by Thabit et al., infertile women were also determined to have significantly higher MDA than controls [58]. MDA was also found to be elevated in the follicular fluid of infertile women, particularly those with endometriosis or reduced ovarian reserve [59,60]. Conversely, in the study of Prieto et al., plasma MDA levels were higher in women with tubal-factor infertility, male-factor infertility, and in the healthy egg donor group compared with patients with endometriosis; however, the difference was not statistically significant [61].

### 4.5. Total Antioxidant Capacity as an Oxidative Stress Biomarker

T-AOC (total antioxidant capacity) reflects the combined action of all antioxidants in biological samples, providing an integrated measure of antioxidant defense [21]. T-AOC is assessed using several laboratory methods, each with its own advantages and limitations. Common assays include FRAP (ferric reducing antioxidant power), ORAC (oxygen radical absorbance capacity), and others, which evaluate the ability of antioxidants to counteract specific oxidant molecules [22,62]. While T-AOC provides a useful overview of antioxidant status, it does not capture the activity of enzymatic antioxidants or the complex interactions of antioxidants in the body. Therefore, T-AOC is best interpreted alongside other clinical and biochemical markers [22].

Clinically, higher dietary or plasma T-AOC is generally associated with a reduced risk of chronic diseases, including cardiovascular disease, type 2 diabetes, and all-cause mortality [63,64,65]. Diets rich in fruits, vegetables, and other plant-based foods tend to have higher T-AOC, and these foods are recommended for their potential protective effects against oxidative stress-related diseases [63,65].

### 4.6. T-AOC as a Marker of Oxidative Stress in Unexplained Infertility

T-AOC measures the overall antioxidant defense in the body. However, only a limited number of studies have investigated T-AOC levels in women with unexplained infertility. In a 2008 study by Appasamy et al., which analyzed plasma and follicular fluid T-AOC among women with various infertility etiologies (including unexplained infertility), no significant association was found between T-AOC levels and the underlying cause of infertility [66]. In 2020, Varnagy et al. published an article revealing a finding that women without endometriosis had higher levels of serum and follicular fluid T-AOC compared with women with endometriosis, complementing our statement that endometriosis contributes to higher levels of OS and, therefore, lower levels of T-AOC may be found for patients with endometriosis [67]. However, in a study published by Zaha et al. in 2023, no significant differences in MDA and T-AOC concentrations in serum and follicular fluid were observed between women undergoing IVF due to female-factor infertility and those with male-factor infertility [68]. In the results of our study, a slight tendency for increasing T-AOC was observed after antioxidant supplementation; however, this finding is noteworthy given the limited number of studies investigating T-AOC levels in women with unexplained infertility, and, to the best of our knowledge, this is the first study where the tendency of increasing T-AOC after antioxidant supplementation was observed.

However, to obtain a more comprehensive picture of oxidative damage for patients, more detailed assessments than the evaluation of a sole biomarker are required, particularly in complex conditions such as unexplained infertility. According to a systematic literature review by Drejza et al. (2022) of biomarkers of OS in various conditions in the field of obstetrics and gynecology, a total of 26 oxidative stress markers were identified, with most studies focusing on five markers, particularly MDA and T-AOC [23]. The authors conclude that there are no universal parameters for assessing oxidative stress in female reproductive disorders and that combining biomarkers may provide a more informative and reliable evaluation [23]. Given the complexity of oxidative stress [11], its assessment benefits from approaches that reflect multiple, complementary biological processes. Combining MDA and T-AOC provides a more comprehensive assessment of oxidative stress, as MDA reflects lipid peroxidation-related oxidative damage [55], while T-AOC represents overall antioxidant defense capacity [24]. Their combined evaluation captures both oxidative injury and compensatory antioxidant responses, improving biological interpretation and clinical relevance.

### 4.7. The Benefits of Antioxidant Therapy

One of the most effective strategies for reducing oxidative stress (OS) in the body is the use of antioxidants. Antioxidants neutralize reactive oxygen species, protecting cells from lipid, protein, and DNA damage [69]. In a randomized controlled trial by Gong et al. (2020), lifestyle and environmental modifications combined with antioxidant supplementation during assisted reproductive technology (ART) procedures were proven to improve fertilization rates and embryo quality [70].

Dietary antioxidants include vitamins A, C, and E; resveratrol; minerals such as selenium and zinc; flavonoids; omega-3 fatty acids; and coenzyme Q10 [69,71]. Foods rich in antioxidants include fruits (especially berries, apples, citrus fruits, and grapes), vegetables (leafy greens, cruciferous vegetables, carrots, tomatoes), nuts, seeds, whole grains, and legumes. Many herbs and spices (turmeric, cinnamon, oregano), mushrooms, dark chocolate, green tea, coffee, and red wine are also natural antioxidant sources [72,73,74,75,76]. Espino et al. found that melatonin supplementation, aimed at reducing OS levels, also had beneficial effects in restoring redox balance and improving oocyte function and embryo quality in women undergoing infertility treatment [77]. Newly emerging antioxidant therapies are also being developed to regulate OS. These include synthetic antioxidants, nanoparticles, inhibitors of advanced glycation end-products (AGEs) and their receptors (RAGE), and single-atom nanoenzymes that target specific oxidative pathways to reduce OS levels in the body [7,71].

Moreover, in a comprehensive review by Reddy et al., it was noted that nanoenzymes—particularly those based on single-atom catalysts (e.g., Mn, Pt, Co, Zn)—can scavenge both reactive oxygen (ROS) and nitrogen species (RNS). Compounds that inhibit AGE formation or break pre-existing AGE crosslinks may also help manage OS and its related disorders [7]. RAGE inhibition reduces OS-mediated signaling cascades. Dietary polyphenols can scavenge ROS, suppress reactive dicarbonyl compound formation, and modulate the AGE–RAGE axis, thereby preventing diseases linked to elevated OS [7].

In our study, the participants were taking antioxidant supplements containing vitamin E, zinc, coenzyme Q10 (CoQ10), and selenium. These antioxidants were selected due to their complementary and well-documented roles in modulating oxidative stress, supporting cellular antioxidant defense mechanisms, and contributing to reproductive function.

Vitamin E exerts multiple beneficial effects on female reproductive health. It contributes to the improvement of endometrial thickness and helps maintain balance between oxidative and antioxidative processes. In addition, vitamin E reduces inflammation [78]. Zinc is essential for proper follicle growth and development, as it contributes to DNA methylation, steroidogenesis, and transcriptional silencing, all of which are required for producing a competent oocyte. Zinc regulates meiotic spindle formation, cytoskeleton and asymmetric cell division and plays a critical role in buffering oxidative stress during oocyte maturation and early embryogenesis, thereby protecting the developing gamete and embryo from ROS-induced damage. At fertilization, zinc participates in egg activation and *Zona pellucida* hardening, processes that prevent polyspermy and support normal zygote formation [79]. CoQ10 improves oocyte quality by promoting energy production, modulating antioxidant gene expression levels, and involvement in various signaling pathways that inhibit oxidative stress and apoptosis [80]. A study published in 2023 by Maddahi et al. demonstrated that supplementation with vitamin E and coenzyme Q10 resulted in significantly higher maturation and cleavage rates compared with coenzyme Q10 combined with vitamin C [81]. The antioxidant role of selenium might alleviate damage caused by follicular fluid oxidative stress [82]. Moreover, according to a study by Satiyeh FD, supplementation with selenium and vitamin E together can increase AMH, antral follicle count and mean ovarian volume in women with occult premature ovarian insufficiency [83].

### 4.8. Limitations

An important limitation of this study is the absence of a control group of fertile women. Consequently, the interpretation of oxidative stress marker levels is restricted to within-subject longitudinal changes rather than comparisons with a reference population, and the absence of a control group precludes causal inference regarding treatment efficacy. Nevertheless, our findings are consistent with those reported in comparable studies, in which oxidative stress levels—based on the assessed biomarkers—were likewise found to be elevated. These comparisons are discussed in detail in the Discussion Section.

Also, a limitation of our study is that only two oxidative stress biomarkers were assessed. However, the evaluation of OS in female infertility remains challenging, as no universally accepted or standardized biomarker panel exists. While the inclusion of a broader panel could provide a more comprehensive characterization of OS status, the biomarkers selected in our study reflect complementary aspects of OS, capturing both oxidative damage and overall antioxidant capacity, and therefore provide meaningful insight into the oxidative balance in our study population.

Moreover, when assessing oxidative stress levels by measuring MDA it is important to take into consideration that serum MDA levels may be influenced by several additional factors, such as the lipid content of the blood at the time of sampling. Higher circulating lipid concentrations can enhance lipid peroxidation processes, leading to an artificially elevated MDA concentration [84]. Moreover, as MDA is typically measured in plasma or serum, it may not fully reflect the oxidative stress status within specific target organs, such as the brain, heart, liver, and kidneys, that are primarily affected in certain OS-driven pathological conditions [85]. Additionally, the concentration of MDA can be affected by the use of certain medications, such as statins, which are known to reduce lipid peroxidation and thereby lower MDA levels [86], and metformin, which has been demonstrated to have a concentration-dependent ability to impede the formation of malondialdehyde (MDA) and other lipid peroxidation products in both in vitro and in vivo models [87,88].

Total antioxidant capacity biomarker may also be influenced by additional factors, such as participants’ dietary antioxidant intake, including consumption of fruits, vegetables, and other antioxidant-rich foods [63,89]. Therefore, variations in baseline diet, as well as potential dietary changes during the supplementation period, may have independently influenced both T-AOC and MDA levels. The absence of dietary data limits our ability to control for this potential confounding factor and may have influenced the observed associations between oxidative stress markers and study outcomes.

Another limitation in assessing oxidative stress through MDA levels is the lack of universally accepted reference values in both scientific literature and clinical practice. Although several studies have attempted to define reference ranges for plasma or serum MDA, the reported values vary widely due to differences in population characteristics, analytical methods, and sample handling procedures. For example, in 1997, Nielsen et al. published a large study in Denmark reporting a reference interval for plasma MDA of 0.36–1.24 μmol/L (0.025 and 0.975 fractiles) in adults aged 20–79, with differences associated with gender and lifestyle factors such as smoking and alcohol consumption [90].

Currently, there is limited and heterogeneous literature regarding the clinical relevance of T-AOC in the context of female infertility, which complicates direct comparison and interpretation of results. In our study, changes in T-AOC were relatively modest and did not demonstrate strong discriminatory power between groups, revealing only tendencies rather than distinct patterns. It is plausible that assessment of a broader panel of oxidative stress-related biomarkers could have enabled identification of more robust patterns and provided clearer insight into redox alterations associated with infertility. Nevertheless, T-AOC reflects the cumulative effect of multiple antioxidant mechanisms and may capture aspects of redox balance not reflected by individual oxidative damage markers alone. Therefore, inclusion of this biomarker within a comprehensive oxidative stress panel may be essential to fully elucidate its potential role in assessing oxidative damage and overall antioxidant capacity. The evidence regarding the advantages and limitations of OS biomarkers assessed in blood and follicular fluid, including MDA and T-AOC, remains inconsistent [60,67]. While biomarkers assessed in follicular fluid are more closely associated with fertility outcomes in women with endometriosis, plasma and serum markers are considered mainly to reflect the overall clinical condition and may have limited prognostic value for infertility-specific conditions [60]. These observations underscore the need for a broader assessment of antioxidant biomarkers in blood to facilitate more accessible diagnostics, as well as for the identification of optimal combinations of oxidative stress biomarkers, given the absence of a universal biomarker panel [23]. Incorporation of additional biomarkers indicative of oxidative DNA damage could also be beneficial.

## 5. Conclusions

In the present study, women with unexplained infertility demonstrated baseline serum MDA and T-AOC levels that are consistent with previously published evidence indicating an imbalance between oxidative damage and antioxidant defense in this population. The observed median baseline MDA concentration reflects increased lipid peroxidation and supports the concept of heightened systemic oxidative stress in women with unexplained infertility, as reported by multiple earlier studies. In parallel, baseline T-AOC values, while less extensively studied in unexplained infertility, suggest a limited compensatory antioxidant capacity and highlight the heterogeneity and complexity of oxidative stress regulation in female reproductive disorders.

Targeted antioxidant supplementation was associated with a reduction in serum MDA levels in women with unexplained infertility, suggesting a decrease in lipid peroxidation and reduced systemic oxidative damage. In contrast, changes in T-AOC were modest and variable, suggesting that global antioxidant capacity may respond less predictably to short-term supplementation or may be influenced by additional regulatory mechanisms beyond direct antioxidant intake. Notably, to the best of our knowledge, this study is the first to report a tendency toward increased T-AOC following antioxidant therapy in women with unexplained infertility, suggesting that systemic antioxidant capacity may be modifiable in this population.

The response to antioxidant therapy varied according to underlying comorbidities, highlighting disease-specific oxidative stress profiles. Women with autoimmune conditions exhibited a less pronounced decrease in oxidative stress following antioxidant therapy, which may reflect the complex immunological and multifactorial etiopathogenesis of these conditions, in which modulation of oxidative stress alone may be insufficient. Conversely, women with endometriosis demonstrated higher baseline MDA levels, consistent with the oxidative stress-driven pathophysiology of the disease, and experienced a statistically significant reduction in oxidative stress following the selected combined antioxidant supplementation, as reflected by the assessed biomarkers, suggesting a greater responsiveness in this subgroup.

Collectively, these findings suggest the potential utility of MDA as a sensitive biomarker for monitoring modulation of oxidative stress, while emphasizing the limited standalone diagnostic and prognostic value of T-AOC and the need for combined biomarker approaches to more accurately characterize oxidative imbalance in female infertility. As, to the best of our knowledge, no previous studies have evaluated changes in T-AOC following antioxidant treatment, the present results may provide a basis for future, more comprehensive investigations.

Importantly, this study demonstrated that combined supplementation with vitamin E, zinc, coenzyme Q10, and selenium may be effective in reducing oxidative stress in women with unexplained infertility, particularly in those with endometriosis, where such interventions may contribute to mitigating the negative impact of oxidative stress on reproductive outcomes.

These results emphasize the importance of stratified, biomarker-guided approaches when considering antioxidant interventions and provide a rationale for future studies into incorporate broader oxidative stress panels and reproductive outcome measures.

## Figures and Tables

**Table 1:** Description of inclusion and exclusion criteria.

Category	List of Criteria
Inclusion criteria	Consent to participate
	Female sex, 18–43 years old
	No clinical signs of menopause
	No current pregnancy or breastfeeding
	No oncologic conditions (current or previous)
	Primary infertility: no previously confirmed pregnancies
	Unexplained infertility: diagnosis established after excluding anatomical, hormonal, infectious, and other causes of infertility
Exclusion criteria	Established causes of infertility confirmed: male-factor infertility, anatomic abnormalities or physical blockage of female reproductive tract, etc.
	Severe comorbidities impairing health/reproductive function Confirmed chronic diseases, which are not controlled or in remission Vulnerable individuals *

* whose health condition precludes them from being considered capable of rationally evaluating their own interests; children; students when participation in biomedical research is related to their studies; people living in social care institutions; soldiers during their actual military service; patients in healthcare institutions undergoing biomedical investigations; employees subordinate to the researcher; people in prisons or other places of deprivation of liberty.

**Table 2:** Distribution of oxidative stress markers MDA and T-AOC in blood serum across chronic disease and autoimmune disorder subgroups as well as the overall study group. The *p*-value indicates the statistical significance of differences between groups, calculated using the Mann–Whitney U test.

Group	Age (Years), Median	MDA1 (ng/mL), Median	MDA2 (ng/mL), Median	T-AOC1 (U/mL), Median	T-AOC2 (U/mL), Median
**Chronic Diseases** No comorbidities (n = 15) With comorbidities (n = 15) *p*-value					
	34 34	304.9 223.5 0.17	188.2 167.9 0.48	24.1 23.7 0.65	26.4 24.5 0.36
**Autoimmune Diseases** No comorbidities (n = 24) With comorbidities (n = 6) *p*-value					
	34 34	246.6 193.1 0.37	182.2 160.3 0.63	22.8 22.0 0.67	24.9 28.8 0.37
**Overall (n = 30)**	34	228.2	173.9	23.9	26.2

**Table 3:** Comparison of oxidative stress biomarkers between individuals with and without most prevalent comorbidities in our study group.

Disease	Group	n	MDA1, ng/mL	MDA2, ng/mL	MDA *p*-Value	T-AOC1, U/mL	T-AOC2, U/mL	T-AOC *p*-Value
Allergic rhinitis	N	25	261.56	188.2	<0.001	24.14	26.38	0.325
	Y	5	149.26	150.3	1.000	22.82	9.72	0.312
Allergic conjunctivitis	N	27	229.91	177.53	<0.001	23.69	26.38	0.400
	Y	3	149.26	140.29	0.750	31.56	9.72	0.250
Atopic dermatitis	N	24	245.73	182.22	<0.001	23.91	25.71	0.705
	Y	6	189.23	148.27	0.094	22.25	29.3	1.000
Endometriosis	N	17	210.38	156.26	<0.001	23.69	26.38	0.818
	Y	13	283.77	221.46	0.005	25.37	26.09	0.893
Bronchial asthma	N	24	224.99	173.94	<0.001	23.25	26.62	0.208
	Y	6	256.84	163.6	0.156	30.61	22.23	0.219
Treated hypothyroidism	N	27	261.56	186.91	<0.001	23.69	26.38	0.500
	Y	3	162.76	108.91	0.250	24.32	14.66	0.500

Results are presented as medians for participants with the diagnosis (Y) and without the diagnosis (N). Within each subgroup, paired comparisons between MDA1 and MDA2 and between T-AOC1 and T-AOC2 were performed using the Wilcoxon signed-rank test. The corresponding *p*-values are provided.

## Data Availability

The original contributions presented in this study are included in the article. Further inquiries can be directed to the corresponding author.
